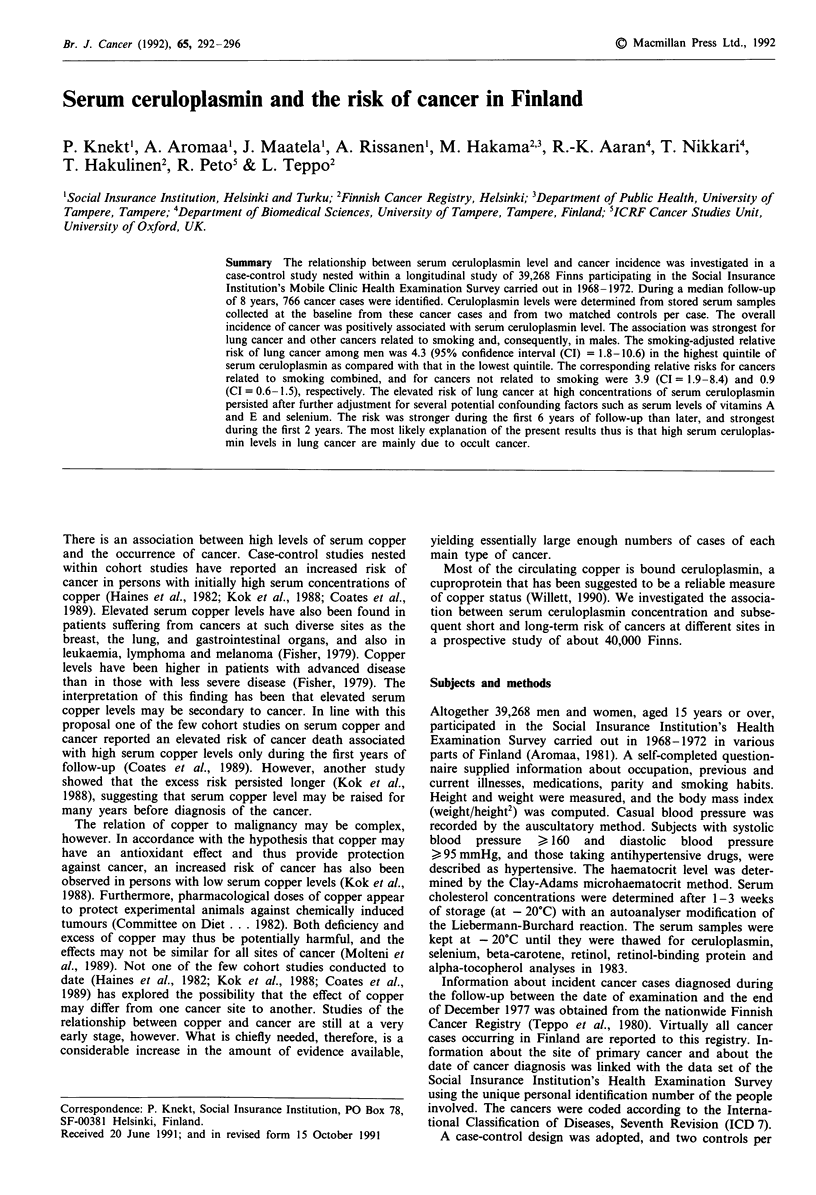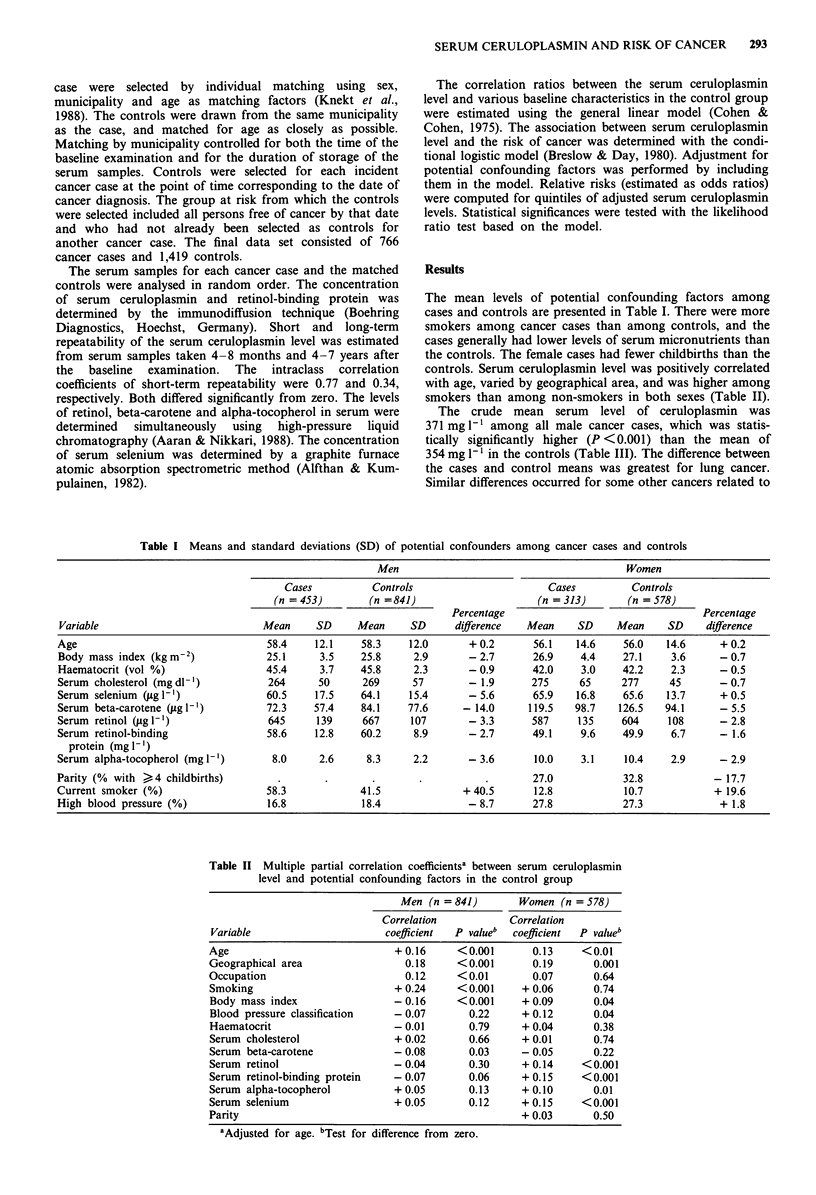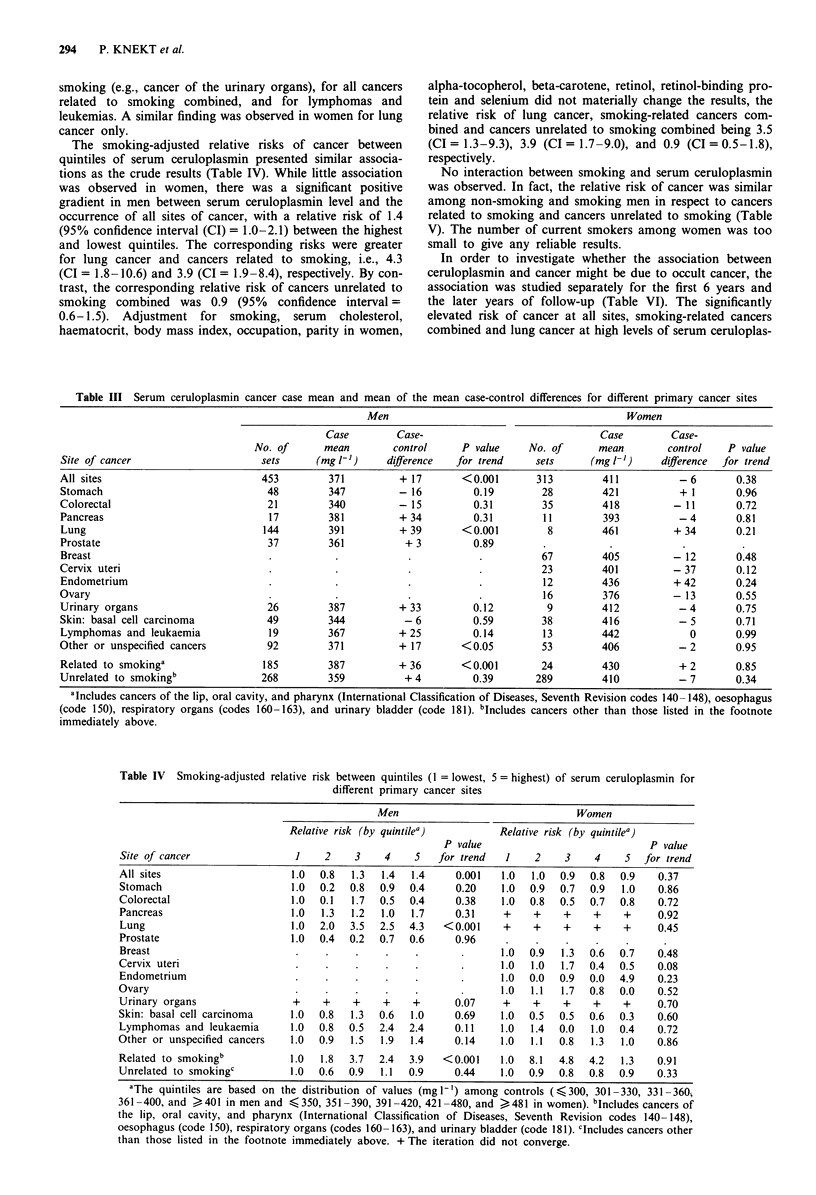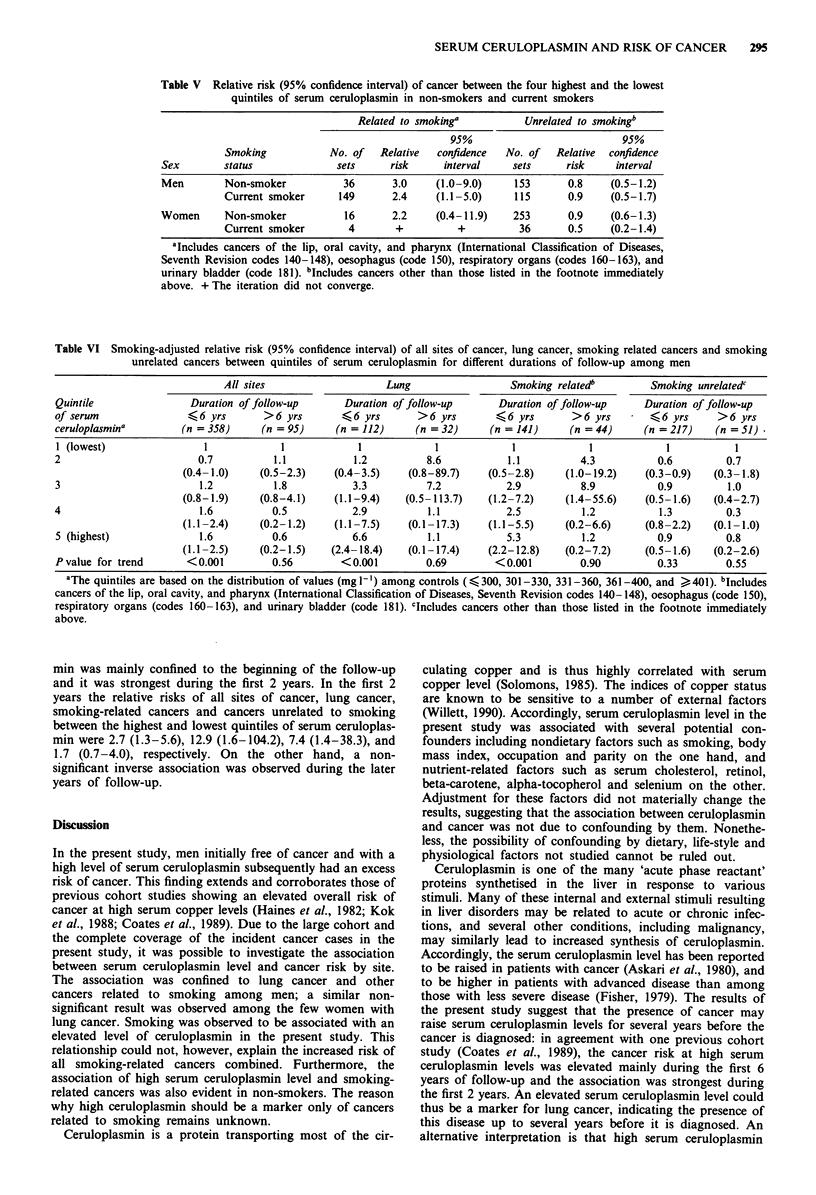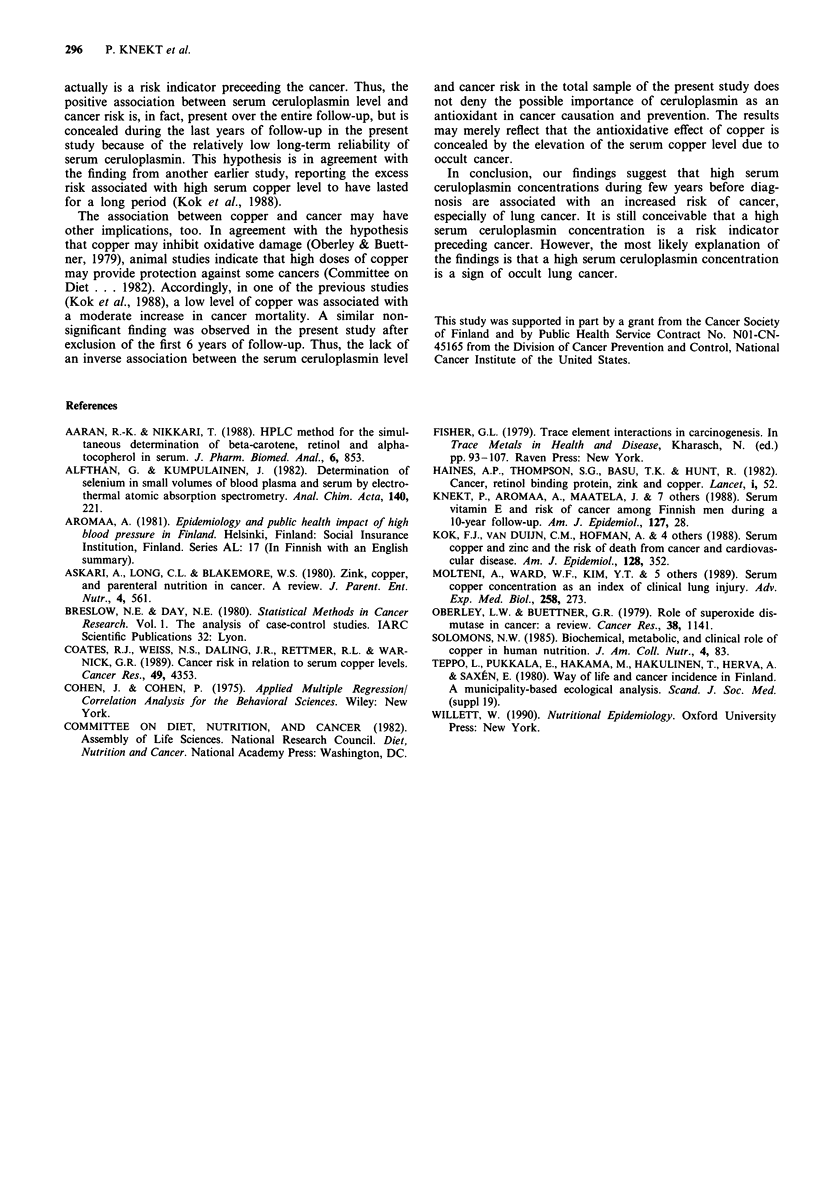# Serum ceruloplasmin and the risk of cancer in Finland.

**DOI:** 10.1038/bjc.1992.58

**Published:** 1992-02

**Authors:** P. Knekt, A. Aromaa, J. Maatela, A. Rissanen, M. Hakama, R. K. Aaran, T. Nikkari, T. Hakulinen, R. Peto, L. Teppo

**Affiliations:** Social Insurance Institution, Helsinki, Finland.

## Abstract

The relationship between serum ceruloplasmin level and cancer incidence was investigated in a case-control study nested within a longitudinal study of 39,268 Finns participating in the Social Insurance Institution's Mobile Clinic Health Examination Survey carried out in 1968-1972. During a median follow-up of 8 years, 766 cancer cases were identified. Ceruloplasmin levels were determined from stored serum samples collected at the baseline from these cancer cases and from two matched controls per case. The overall incidence of cancer was positively associated with serum ceruloplasmin level. The association was strongest for lung cancer and other cancers related to smoking and, consequently, in males. The smoking-adjusted relative risk of lung cancer among men was 4.3 (95% confidence interval (CI) = 1.8-10.6) in the highest quintile of serum ceruloplasmin as compared with that in the lowest quintile. The corresponding relative risks for cancers related to smoking combined, and for cancers not related to smoking were 3.9 (CI = 1.9-8.4) and 0.9 (CI = 0.6-1.5), respectively. The elevated risk of lung cancer at high concentrations of serum ceruloplasmin persisted after further adjustment for several potential confounding factors such as serum levels of vitamins A and E and selenium. The risk was stronger during the first 6 years of follow-up than later, and strongest during the first 2 years. The most likely explanation of the present results thus is that high serum ceruloplasmin levels in lung cancer are mainly due to occult cancer.


Br. J. Cancer (1992), 65, 292-296 © Macmillan Press Ltd., 1992

### Serum ceruloplasmin and the risk of cancer in Finland

P. Knekt[1], A. Aromaa[1], J. Maatela[1], A. Rissanen[1], M. Hakama[2,3], R.-K. Aaran[4], T. Nikkari[4], T. Hakulinen[2], R. Peto[5] & L. Teppo[2]

[1]*Social Insurance Institution, Helsinki and Turku;* [2]*Finnish Cancer Registry, Helsinki;* [3]*Department of Public Health, University of Tampere, Tampere;* [4]*Department of Biomedical Sciences, University of Tampere, Tampere, Finland;* [5]*ICRF Cancer Studies Unit, University of Oxford, UK.*

**Summary** The relationship between serum ceruloplasmin level and cancer incidence was investigated in a case-control study nested within a longitudinal study of 39,268 Finns participating in the Social Insurance Institution's Mobile Clinic Health Examination Survey carried out in 1968-1972. During a median follow-up of 8 years, 766 cancer cases were identified. Ceruloplasmin levels were determined from stored serum samples collected at the baseline from these cancer cases and from two matched controls per case. The overall incidence of cancer was positively associated with serum ceruloplasmin level. The association was strongest for lung cancer and other cancers related to smoking and, consequently, in males. The smoking-adjusted relative risk of lung cancer among men was 4.3 (95% confidence interval (CI) = 1.8-10.6) in the highest quintile of serum ceruloplasmin as compared with that in the lowest quintile. The corresponding relative risks for cancers related to smoking combined, and for cancers not related to smoking were 3.9 (CI = 1.9-8.4) and 0.9 (CI = 0.6-1.5), respectively. The elevated risk of lung cancer at high concentrations of serum ceruloplasmin persisted after further adjustment for several potential confounding factors such as serum levels of vitamins A and E and selenium. The risk was stronger during the first 6 years of follow-up than later, and strongest during the first 2 years. The most likely explanation of the present results thus is that high serum ceruloplasmin levels in lung cancer are mainly due to occult cancer.

There is an association between high levels of serum copper and the occurrence of cancer. Case-control studies nested within cohort studies have reported an increased risk of cancer in persons with initially high serum concentrations of copper (Haines *et al.*, 1982; Kok *et al.*, 1988; Coates *et al.*, 1989). Elevated serum copper levels have also been found in patients suffering from cancers at such diverse sites as the breast, the lung, and gastrointestinal organs, and also in leukaemia, lymphoma and melanoma (Fisher, 1979). Copper levels have been higher in patients with advanced disease than in those with less severe disease (Fisher, 1979). The interpretation of this finding has been that elevated serum copper levels may be secondary to cancer. In line with this proposal one of the few cohort studies on serum copper and cancer reported an elevated risk of cancer death associated with high serum copper levels only during the first years of follow-up (Coates *et al.*, 1989). However, another study showed that the excess risk persisted longer (Kok *et al.*, 1988), suggesting that serum copper level may be raised for many years before diagnosis of the cancer.

The relation of copper to malignancy may be complex, however. In accordance with the hypothesis that copper may have an antioxidant effect and thus provide protection against cancer, an increased risk of cancer has also been observed in persons with low serum copper levels (Kok *et al.*, 1988). Furthermore, pharmacological doses of copper appear to protect experimental animals against chemically induced tumours (Committee on Diet . . . 1982). Both deficiency and excess of copper may thus be potentially harmful, and the effects may not be similar for all sites of cancer (Molteni *et al.*, 1989). Not one of the few cohort studies conducted to date (Haines *et al.*, 1982; Kok *et al.*, 1988; Coates *et al.*, 1989) has explored the possibility that the effect of copper may differ from one cancer site to another. Studies of the relationship between copper and cancer are still at a very early stage, however. What is chiefly needed, therefore, is a considerable increase in the amount of evidence available, yielding essentially large enough numbers of cases of each main type of cancer.

Most of the circulating copper is bound ceruloplasmin, a cuproprotein that has been suggested to be a reliable measure of copper status (Willett, 1990). We investigated the association between serum ceruloplasmin concentration and subsequent short and long-term risk of cancers at different sites in a prospective study of about 40,000 Finns.

### Subjects and methods

Altogether 39,268 men and women, aged 15 years or over, participated in the Social Insurance Institution's Health Examination Survey carried out in 1968-1972 in various parts of Finland (Aromaa, 1981). A self-completed questionnaire supplied information about occupation, previous and current illnesses, medications, parity and smoking habits. Height and weight were measured, and the body mass index (weight/height$^2$) was computed. Casual blood pressure was recorded by the auscultatory method. Subjects with systolic blood pressure $\geqslant 160$ and diastolic blood pressure $\geqslant 95$ mmHg, and those taking antihypertensive drugs, were described as hypertensive. The haematocrit level was determined by the Clay-Adams microhaematocrit method. Serum cholesterol concentrations were determined after 1-3 weeks of storage (at $-20$°C) with an autoanalyser modification of the Liebermann-Burchard reaction. The serum samples were kept at $-20$°C until they were thawed for ceruloplasmin, selenium, beta-carotene, retinol, retinol-binding protein and alpha-tocopherol analyses in 1983.

Information about incident cancer cases diagnosed during the follow-up between the date of examination and the end of December 1977 was obtained from the nationwide Finnish Cancer Registry (Teppo *et al.*, 1980). Virtually all cancer cases occurring in Finland are reported to this registry. Information about the site of primary cancer and about the date of cancer diagnosis was linked with the data set of the Social Insurance Institution's Health Examination Survey using the unique personal identification number of the people involved. The cancers were coded according to the International Classification of Diseases, Seventh Revision (ICD 7).

A case-control design was adopted, and two controls per

Correspondence: P. Knekt, Social Insurance Institution, PO Box 78, SF-00381 Helsinki, Finland.
Received 20 June 1991; and in revised form 15 October 1991

case were selected by individual matching using sex, municipality and age as matching factors (Knekt *et al.*, 1988). The controls were drawn from the same municipality as the case, and matched for age as closely as possible. Matching by municipality controlled for both the time of the baseline examination and for the duration of storage of the serum samples. Controls were selected for each incident cancer case at the point of time corresponding to the date of cancer diagnosis. The group at risk from which the controls were selected included all persons free of cancer by that date and who had not already been selected as controls for another cancer case. The final data set consisted of 766 cancer cases and 1,419 controls.

The serum samples for each cancer case and the matched controls were analysed in random order. The concentration of serum ceruloplasmin and retinol-binding protein was determined by the immunodiffusion technique (Boehring Diagnostics, Hoechst, Germany). Short and long-term repeatability of the serum ceruloplasmin level was estimated from serum samples taken 4–8 months and 4–7 years after the baseline examination. The intraclass correlation coefficients of short-term repeatability were 0.77 and 0.34, respectively. Both differed significantly from zero. The levels of retinol, beta-carotene and alpha-tocopherol in serum were determined simultaneously using high-pressure liquid chromatography (Aaran & Nikkari, 1988). The concentration of serum selenium was determined by a graphite furnace atomic absorption spectrometric method (Alfthan & Kumpulainen, 1982).

The correlation ratios between the serum ceruloplasmin level and various baseline characteristics in the control group were estimated using the general linear model (Cohen & Cohen, 1975). The association between serum ceruloplasmin level and the risk of cancer was determined with the conditional logistic model (Breslow & Day, 1980). Adjustment for potential confounding factors was performed by including them in the model. Relative risks (estimated as odds ratios) were computed for quintiles of adjusted serum ceruloplasmin levels. Statistical significances were tested with the likelihood ratio test based on the model.

### Results

The mean levels of potential confounding factors among cases and controls are presented in Table I. There were more smokers among cancer cases than among controls, and the cases generally had lower levels of serum micronutrients than the controls. The female cases had fewer childbirths than the controls. Serum ceruloplasmin level was positively correlated with age, varied by geographical area, and was higher among smokers than among non-smokers in both sexes (Table II).

The crude mean serum level of ceruloplasmin was 371 mg $l^{-1}$ among all male cancer cases, which was statistically significantly higher ($P < 0.001$) than the mean of 354 mg $l^{-1}$ in the controls (Table III). The difference between the cases and control means was greatest for lung cancer. Similar differences occurred for some other cancers related to

**Table I** Means and standard deviations (SD) of potential confounders among cancer cases and controls

| | *Men* | | | | | *Women* | | | | |
|---|---|---|---|---|---|---|---|---|---|---|
| | *Cases (n = 453)* | | *Controls (n = 841)* | | | *Cases (n = 313)* | | *Controls (n = 578)* | | |
| *Variable* | *Mean* | *SD* | *Mean* | *SD* | *Percentage difference* | *Mean* | *SD* | *Mean* | *SD* | *Percentage difference* |
| Age | 58.4 | 12.1 | 58.3 | 12.0 | + 0.2 | 56.1 | 14.6 | 56.0 | 14.6 | + 0.2 |
| Body mass index (kg $m^{-2}$) | 25.1 | 3.5 | 25.8 | 2.9 | − 2.7 | 26.9 | 4.4 | 27.1 | 3.6 | − 0.7 |
| Haematocrit (vol %) | 45.4 | 3.7 | 45.8 | 2.3 | − 0.9 | 42.0 | 3.0 | 42.2 | 2.3 | − 0.5 |
| Serum cholesterol (mg $dl^{-1}$) | 264 | 50 | 269 | 57 | − 1.9 | 275 | 65 | 277 | 45 | − 0.7 |
| Serum selenium (μg $l^{-1}$) | 60.5 | 17.5 | 64.1 | 15.4 | − 5.6 | 65.9 | 16.8 | 65.6 | 13.7 | + 0.5 |
| Serum beta-carotene (μg $l^{-1}$) | 72.3 | 57.4 | 84.1 | 77.6 | − 14.0 | 119.5 | 98.7 | 126.5 | 94.1 | − 5.5 |
| Serum retinol (μg $l^{-1}$) | 645 | 139 | 667 | 107 | − 3.3 | 587 | 135 | 604 | 108 | − 2.8 |
| Serum retinol-binding protein (mg $l^{-1}$) | 58.6 | 12.8 | 60.2 | 8.9 | − 2.7 | 49.1 | 9.6 | 49.9 | 6.7 | − 1.6 |
| Serum alpha-tocopherol (mg $l^{-1}$) | 8.0 | 2.6 | 8.3 | 2.2 | − 3.6 | 10.0 | 3.1 | 10.4 | 2.9 | − 2.9 |
| Parity (% with ≥4 childbirths) | . | . | . | . | . | 27.0 | | 32.8 | | − 17.7 |
| Current smoker (%) | 58.3 | | 41.5 | | + 40.5 | 12.8 | | 10.7 | | + 19.6 |
| High blood pressure (%) | 16.8 | | 18.4 | | − 8.7 | 27.8 | | 27.3 | | + 1.8 |

**Table II** Multiple partial correlation coefficients[a] between serum ceruloplasmin level and potential confounding factors in the control group

| | *Men (n = 841)* | | *Women (n = 578)* | |
|---|---|---|---|---|
| *Variable* | *Correlation coefficient* | *P value*[b] | *Correlation coefficient* | *P value*[b] |
| Age | + 0.16 | <0.001 | 0.13 | <0.01 |
| Geographical area | 0.18 | <0.001 | 0.19 | 0.001 |
| Occupation | 0.12 | <0.01 | 0.07 | 0.64 |
| Smoking | + 0.24 | <0.001 | + 0.06 | 0.74 |
| Body mass index | − 0.16 | <0.001 | + 0.09 | 0.04 |
| Blood pressure classification | − 0.07 | 0.22 | + 0.12 | 0.04 |
| Haematocrit | − 0.01 | 0.79 | + 0.04 | 0.38 |
| Serum cholesterol | + 0.02 | 0.66 | + 0.01 | 0.74 |
| Serum beta-carotene | − 0.08 | 0.03 | − 0.05 | 0.22 |
| Serum retinol | − 0.04 | 0.30 | + 0.14 | <0.001 |
| Serum retinol-binding protein | − 0.07 | 0.06 | + 0.15 | <0.001 |
| Serum alpha-tocopherol | + 0.05 | 0.13 | + 0.10 | 0.01 |
| Serum selenium | + 0.05 | 0.12 | + 0.15 | <0.001 |
| Parity | | | + 0.03 | 0.50 |

[a]Adjusted for age. [b]Test for difference from zero.

smoking (e.g., cancer of the urinary organs), for all cancers related to smoking combined, and for lymphomas and leukemias. A similar finding was observed in women for lung cancer only.

The smoking-adjusted relative risks of cancer between quintiles of serum ceruloplasmin presented similar associations as the crude results (Table IV). While little association was observed in women, there was a significant positive gradient in men between serum ceruloplasmin level and the occurrence of all sites of cancer, with a relative risk of 1.4 (95% confidence interval (CI) = 1.0–2.1) between the highest and lowest quintiles. The corresponding risks were greater for lung cancer and cancers related to smoking, i.e., 4.3 (CI = 1.8–10.6) and 3.9 (CI = 1.9–8.4), respectively. By contrast, the corresponding relative risk of cancers unrelated to smoking combined was 0.9 (95% confidence interval = 0.6–1.5). Adjustment for smoking, serum cholesterol, haematocrit, body mass index, occupation, parity in women, alpha-tocopherol, beta-carotene, retinol, retinol-binding protein and selenium did not materially change the results, the relative risk of lung cancer, smoking-related cancers combined and cancers unrelated to smoking combined being 3.5 (CI = 1.3–9.3), 3.9 (CI = 1.7–9.0), and 0.9 (CI = 0.5–1.8), respectively.

No interaction between smoking and serum ceruloplasmin was observed. In fact, the relative risk of cancer was similar among non-smoking and smoking men in respect to cancers related to smoking and cancers unrelated to smoking (Table V). The number of current smokers among women was too small to give any reliable results.

In order to investigate whether the association between ceruloplasmin and cancer might be due to occult cancer, the association was studied separately for the first 6 years and the later years of follow-up (Table VI). The significantly elevated risk of cancer at all sites, smoking-related cancers combined and lung cancer at high levels of serum ceruloplas-

**Table III** Serum ceruloplasmin cancer case mean and mean of the mean case-control differences for different primary cancer sites

| | *Men* | | | | *Women* | | | |
|---|---|---|---|---|---|---|---|---|
| *Site of cancer* | *No. of sets* | *Case mean (mg l⁻¹)* | *Case-control difference* | *P value for trend* | *No. of sets* | *Case mean (mg l⁻¹)* | *Case-control difference* | *P value for trend* |
| All sites | 453 | 371 | + 17 | <0.001 | 313 | 411 | − 6 | 0.38 |
| Stomach | 48 | 347 | − 16 | 0.19 | 28 | 421 | + 1 | 0.96 |
| Colorectal | 21 | 340 | − 15 | 0.31 | 35 | 418 | − 11 | 0.72 |
| Pancreas | 17 | 381 | + 34 | 0.31 | 11 | 393 | − 4 | 0.81 |
| Lung | 144 | 391 | + 39 | <0.001 | 8 | 461 | + 34 | 0.21 |
| Prostate | 37 | 361 | + 3 | 0.89 | . | . | . | . |
| Breast | . | . | . | . | 67 | 405 | − 12 | 0.48 |
| Cervix uteri | . | . | . | . | 23 | 401 | − 37 | 0.12 |
| Endometrium | . | . | . | . | 12 | 436 | + 42 | 0.24 |
| Ovary | . | . | . | . | 16 | 376 | − 13 | 0.55 |
| Urinary organs | 26 | 387 | + 33 | 0.12 | 9 | 412 | − 4 | 0.75 |
| Skin: basal cell carcinoma | 49 | 344 | − 6 | 0.59 | 38 | 416 | − 5 | 0.71 |
| Lymphomas and leukaemia | 19 | 367 | + 25 | 0.14 | 13 | 442 | 0 | 0.99 |
| Other or unspecified cancers | 92 | 371 | + 17 | <0.05 | 53 | 406 | − 2 | 0.95 |
| Related to smoking[a] | 185 | 387 | + 36 | <0.001 | 24 | 430 | + 2 | 0.85 |
| Unrelated to smoking[b] | 268 | 359 | + 4 | 0.39 | 289 | 410 | − 7 | 0.34 |

[a]Includes cancers of the lip, oral cavity, and pharynx (International Classification of Diseases, Seventh Revision codes 140–148), oesophagus (code 150), respiratory organs (codes 160–163), and urinary bladder (code 181). [b]Includes cancers other than those listed in the footnote immediately above.

**Table IV** Smoking-adjusted relative risk between quintiles (1 = lowest, 5 = highest) of serum ceruloplasmin for different primary cancer sites

| | *Men* | | | | | | *Women* | | | | | |
|---|---|---|---|---|---|---|---|---|---|---|---|---|
| | *Relative risk (by quintile[a])* | | | | | | *Relative risk (by quintile[a])* | | | | | |
| *Site of cancer* | *1* | *2* | *3* | *4* | *5* | *P value for trend* | *1* | *2* | *3* | *4* | *5* | *P value for trend* |
| All sites | 1.0 | 0.8 | 1.3 | 1.4 | 1.4 | 0.001 | 1.0 | 1.0 | 0.9 | 0.8 | 0.9 | 0.37 |
| Stomach | 1.0 | 0.2 | 0.8 | 0.9 | 0.4 | 0.20 | 1.0 | 0.9 | 0.7 | 0.9 | 1.0 | 0.86 |
| Colorectal | 1.0 | 0.1 | 1.7 | 0.5 | 0.4 | 0.38 | 1.0 | 0.8 | 0.5 | 0.7 | 0.8 | 0.72 |
| Pancreas | 1.0 | 1.3 | 1.2 | 1.0 | 1.7 | 0.31 | + | + | + | + | + | 0.92 |
| Lung | 1.0 | 2.0 | 3.5 | 2.5 | 4.3 | <0.001 | + | + | + | + | + | 0.45 |
| Prostate | 1.0 | 0.4 | 0.2 | 0.7 | 0.6 | 0.96 | . | . | . | . | . | . |
| Breast | . | . | . | . | . | . | 1.0 | 0.9 | 1.3 | 0.6 | 0.7 | 0.48 |
| Cervix uteri | . | . | . | . | . | . | 1.0 | 1.0 | 1.7 | 0.4 | 0.5 | 0.08 |
| Endometrium | . | . | . | . | . | . | 1.0 | 0.0 | 0.9 | 0.0 | 4.9 | 0.23 |
| Ovary | . | . | . | . | . | . | 1.0 | 1.1 | 1.7 | 0.8 | 0.0 | 0.52 |
| Urinary organs | + | + | + | + | + | 0.07 | + | + | + | + | + | 0.70 |
| Skin: basal cell carcinoma | 1.0 | 0.8 | 1.3 | 0.6 | 1.0 | 0.69 | 1.0 | 0.5 | 0.5 | 0.6 | 0.3 | 0.60 |
| Lymphomas and leukaemia | 1.0 | 0.8 | 0.5 | 2.4 | 2.4 | 0.11 | 1.0 | 1.4 | 0.0 | 1.0 | 0.4 | 0.72 |
| Other or unspecified cancers | 1.0 | 0.9 | 1.5 | 1.9 | 1.4 | 0.14 | 1.0 | 1.1 | 0.8 | 1.3 | 1.0 | 0.86 |
| Related to smoking[b] | 1.0 | 1.8 | 3.7 | 2.4 | 3.9 | <0.001 | 1.0 | 8.1 | 4.8 | 4.2 | 1.3 | 0.91 |
| Unrelated to smoking[c] | 1.0 | 0.6 | 0.9 | 1.1 | 0.9 | 0.44 | 1.0 | 0.9 | 0.8 | 0.8 | 0.9 | 0.33 |

[a]The quintiles are based on the distribution of values (mg l⁻¹) among controls (⩽300, 301–330, 331–360, 361–400, and ⩾401 in men and ⩽350, 351–390, 391–420, 421–480, and ⩾481 in women). [b]Includes cancers of the lip, oral cavity, and pharynx (International Classification of Diseases, Seventh Revision codes 140–148), oesophagus (code 150), respiratory organs (codes 160–163), and urinary bladder (code 181). [c]Includes cancers other than those listed in the footnote immediately above. + The iteration did not converge.

**Table V** Relative risk (95% confidence interval) of cancer between the four highest and the lowest quintiles of serum ceruloplasmin in non-smokers and current smokers

| | | *Related to smoking*[a] | | | *Unrelated to smoking*[b] | | |
|---|---|---|---|---|---|---|---|
| *Sex* | *Smoking status* | *No. of sets* | *Relative risk* | *95% confidence interval* | *No. of sets* | *Relative risk* | *95% confidence interval* |
| Men | Non-smoker | 36 | 3.0 | (1.0–9.0) | 153 | 0.8 | (0.5–1.2) |
| | Current smoker | 149 | 2.4 | (1.1–5.0) | 115 | 0.9 | (0.5–1.7) |
| Women | Non-smoker | 16 | 2.2 | (0.4–11.9) | 253 | 0.9 | (0.6–1.3) |
| | Current smoker | 4 | + | + | 36 | 0.5 | (0.2–1.4) |

[a]Includes cancers of the lip, oral cavity, and pharynx (International Classification of Diseases, Seventh Revision codes 140–148), oesophagus (code 150), respiratory organs (codes 160–163), and urinary bladder (code 181). [b]Includes cancers other than those listed in the footnote immediately above. + The iteration did not converge.

**Table VI** Smoking-adjusted relative risk (95% confidence interval) of all sites of cancer, lung cancer, smoking related cancers and smoking unrelated cancers between quintiles of serum ceruloplasmin for different durations of follow-up among men

| | *All sites* | | *Lung* | | *Smoking related*[b] | | *Smoking unrelated*[c] | |
|---|---|---|---|---|---|---|---|---|
| *Quintile of serum ceruloplasmin*[a] | *Duration of follow-up* ≤*6 yrs (n = 358)* | *>6 yrs (n = 95)* | *Duration of follow-up* ≤*6 yrs (n = 112)* | *>6 yrs (n = 32)* | *Duration of follow-up* ≤*6 yrs (n = 141)* | *>6 yrs (n = 44)* | *Duration of follow-up* ≤*6 yrs (n = 217)* | *>6 yrs (n = 51)* |
| 1 (lowest) | 1 | 1 | 1 | 1 | 1 | 1 | 1 | 1 |
| 2 | 0.7 | 1.1 | 1.2 | 8.6 | 1.1 | 4.3 | 0.6 | 0.7 |
| | (0.4–1.0) | (0.5–2.3) | (0.4–3.5) | (0.8–89.7) | (0.5–2.8) | (1.0–19.2) | (0.3–0.9) | (0.3–1.8) |
| 3 | 1.2 | 1.8 | 3.3 | 7.2 | 2.9 | 8.9 | 0.9 | 1.0 |
| | (0.8–1.9) | (0.8–4.1) | (1.1–9.4) | (0.5–113.7) | (1.2–7.2) | (1.4–55.6) | (0.5–1.6) | (0.4–2.7) |
| 4 | 1.6 | 0.5 | 2.9 | 1.1 | 2.5 | 1.2 | 1.3 | 0.3 |
| | (1.1–2.4) | (0.2–1.2) | (1.1–7.5) | (0.1–17.3) | (1.1–5.5) | (0.2–6.6) | (0.8–2.2) | (0.1–1.0) |
| 5 (highest) | 1.6 | 0.6 | 6.6 | 1.1 | 5.3 | 1.2 | 0.9 | 0.8 |
| | (1.1–2.5) | (0.2–1.5) | (2.4–18.4) | (0.1–17.4) | (2.2–12.8) | (0.2–7.2) | (0.5–1.6) | (0.2–2.6) |
| *P* value for trend | <0.001 | 0.56 | <0.001 | 0.69 | <0.001 | 0.90 | 0.33 | 0.55 |

[a]The quintiles are based on the distribution of values (mg $l^{-1}$) among controls (≤300, 301–330, 331–360, 361–400, and ≥401). [b]Includes cancers of the lip, oral cavity, and pharynx (International Classification of Diseases, Seventh Revision codes 140–148), oesophagus (code 150), respiratory organs (codes 160–163), and urinary bladder (code 181). [c]Includes cancers other than those listed in the footnote immediately above.

min was mainly confined to the beginning of the follow-up and it was strongest during the first 2 years. In the first 2 years the relative risks of all sites of cancer, lung cancer, smoking-related cancers and cancers unrelated to smoking between the highest and lowest quintiles of serum ceruloplasmin were 2.7 (1.3–5.6), 12.9 (1.6–104.2), 7.4 (1.4–38.3), and 1.7 (0.7–4.0), respectively. On the other hand, a non-significant inverse association was observed during the later years of follow-up.

### Discussion

In the present study, men initially free of cancer and with a high level of serum ceruloplasmin subsequently had an excess risk of cancer. This finding extends and corroborates those of previous cohort studies showing an elevated overall risk of cancer at high serum copper levels (Haines *et al*., 1982; Kok *et al*., 1988; Coates *et al*., 1989). Due to the large cohort and the complete coverage of the incident cancer cases in the present study, it was possible to investigate the association between serum ceruloplasmin level and cancer risk by site. The association was confined to lung cancer and other cancers related to smoking among men; a similar non-significant result was observed among the few women with lung cancer. Smoking was observed to be associated with an elevated level of ceruloplasmin in the present study. This relationship could not, however, explain the increased risk of all smoking-related cancers combined. Furthermore, the association of high serum ceruloplasmin level and smoking-related cancers was also evident in non-smokers. The reason why high ceruloplasmin should be a marker only of cancers related to smoking remains unknown.

Ceruloplasmin is a protein transporting most of the circulating copper and is thus highly correlated with serum copper level (Solomons, 1985). The indices of copper status are known to be sensitive to a number of external factors (Willett, 1990). Accordingly, serum ceruloplasmin level in the present study was associated with several potential confounders including nondietary factors such as smoking, body mass index, occupation and parity on the one hand, and nutrient-related factors such as serum cholesterol, retinol, beta-carotene, alpha-tocopherol and selenium on the other. Adjustment for these factors did not materially change the results, suggesting that the association between ceruloplasmin and cancer was not due to confounding by them. Nonetheless, the possibility of confounding by dietary, life-style and physiological factors not studied cannot be ruled out.

Ceruloplasmin is one of the many 'acute phase reactant' proteins synthetised in the liver in response to various stimuli. Many of these internal and external stimuli resulting in liver disorders may be related to acute or chronic infections, and several other conditions, including malignancy, may similarly lead to increased synthesis of ceruloplasmin. Accordingly, the serum ceruloplasmin level has been reported to be raised in patients with cancer (Askari *et al*., 1980), and to be higher in patients with advanced disease than among those with less severe disease (Fisher, 1979). The results of the present study suggest that the presence of cancer may raise serum ceruloplasmin levels for several years before the cancer is diagnosed: in agreement with one previous cohort study (Coates *et al*., 1989), the cancer risk at high serum ceruloplasmin levels was elevated mainly during the first 6 years of follow-up and the association was strongest during the first 2 years. An elevated serum ceruloplasmin level could thus be a marker for lung cancer, indicating the presence of this disease up to several years before it is diagnosed. An alternative interpretation is that high serum ceruloplasmin actually is a risk indicator preceeding the cancer. Thus, the positive association between serum ceruloplasmin level and cancer risk is, in fact, present over the entire follow-up, but is concealed during the last years of follow-up in the present study because of the relatively low long-term reliability of serum ceruloplasmin. This hypothesis is in agreement with the finding from another earlier study, reporting the excess risk associated with high serum copper level to have lasted for a long period (Kok *et al.*, 1988).

The association between copper and cancer may have other implications, too. In agreement with the hypothesis that copper may inhibit oxidative damage (Oberley & Buettner, 1979), animal studies indicate that high doses of copper may provide protection against some cancers (Committee on Diet . . . 1982). Accordingly, in one of the previous studies (Kok *et al.*, 1988), a low level of copper was associated with a moderate increase in cancer mortality. A similar non-significant finding was observed in the present study after exclusion of the first 6 years of follow-up. Thus, the lack of an inverse association between the serum ceruloplasmin level and cancer risk in the total sample of the present study does not deny the possible importance of ceruloplasmin as an antioxidant in cancer causation and prevention. The results may merely reflect that the antioxidative effect of copper is concealed by the elevation of the serum copper level due to occult cancer.

In conclusion, our findings suggest that high serum ceruloplasmin concentrations during few years before diagnosis are associated with an increased risk of cancer, especially of lung cancer. It is still conceivable that a high serum ceruloplasmin concentration is a risk indicator preceding cancer. However, the most likely explanation of the findings is that a high serum ceruloplasmin concentration is a sign of occult lung cancer.

This study was supported in part by a grant from the Cancer Society of Finland and by Public Health Service Contract No. N01-CN-45165 from the Division of Cancer Prevention and Control, National Cancer Institute of the United States.

### References

AARAN, R.-K. & NIKKARI, T. (1988). HPLC method for the simultaneous determination of beta-carotene, retinol and alpha-tocopherol in serum. *J. Pharm. Biomed. Anal.*, **6**, 853.

ALFTHAN, G. & KUMPULAINEN, J. (1982). Determination of selenium in small volumes of blood plasma and serum by electrothermal atomic absorption spectrometry. *Anal. Chim. Acta*, **140**, 221.

AROMAA, A. (1981). *Epidemiology and public health impact of high blood pressure in Finland*. Helsinki, Finland: Social Insurance Institution, Finland. Series AL: 17 (In Finnish with an English summary).

ASKARI, A., LONG, C.L. & BLAKEMORE, W.S. (1980). Zink, copper, and parenteral nutrition in cancer. A review. *J. Parent. Ent. Nutr.*, **4**, 561.

BRESLOW, N.E. & DAY, N.E. (1980). *Statistical Methods in Cancer Research*. Vol. 1. The analysis of case-control studies. IARC Scientific Publications 32: Lyon.

COATES, R.J., WEISS, N.S., DALING, J.R., RETTMER, R.L. & WARNICK, G.R. (1989). Cancer risk in relation to serum copper levels. *Cancer Res.*, **49**, 4353.

COHEN, J. & COHEN, P. (1975). *Applied Multiple Regression/Correlation Analysis for the Behavioral Sciences*. Wiley: New York.

COMMITTEE ON DIET, NUTRITION, AND CANCER (1982). Assembly of Life Sciences. National Research Council. *Diet, Nutrition and Cancer*. National Academy Press: Washington, DC.

FISHER, G.L. (1979). Trace element interactions in carcinogenesis. In *Trace Metals in Health and Disease*, Kharasch, N. (ed.) pp. 93–107. Raven Press: New York.

HAINES, A.P., THOMPSON, S.G., BASU, T.K. & HUNT, R. (1982). Cancer, retinol binding protein, zink and copper. *Lancet*, **i**, 52.

KNEKT, P., AROMAA, A., MAATELA, J. & 7 others (1988). Serum vitamin E and risk of cancer among Finnish men during a 10-year follow-up. *Am. J. Epidemiol.*, **127**, 28.

KOK, F.J., VAN DUIJN, C.M., HOFMAN, A. & 4 others (1988). Serum copper and zinc and the risk of death from cancer and cardiovascular disease. *Am. J. Epidemiol.*, **128**, 352.

MOLTENI, A., WARD, W.F., KIM, Y.T. & 5 others (1989). Serum copper concentration as an index of clinical lung injury. *Adv. Exp. Med. Biol.*, **258**, 273.

OBERLEY, L.W. & BUETTNER, G.R. (1979). Role of superoxide dismutase in cancer: a review. *Cancer Res.*, **38**, 1141.

SOLOMONS, N.W. (1985). Biochemical, metabolic, and clinical role of copper in human nutrition. *J. Am. Coll. Nutr.*, **4**, 83.

TEPPO, L., PUKKALA, E., HAKAMA, M., HAKULINEN, T., HERVA, A. & SAXÉN, E. (1980). Way of life and cancer incidence in Finland. A municipality-based ecological analysis. *Scand. J. Soc. Med.* (suppl 19).

WILLETT, W. (1990). *Nutritional Epidemiology*. Oxford University Press: New York.